# Hypoparathyroidism: update of guidelines from the 2022 International Task Force

**DOI:** 10.20945/2359-3997000000549

**Published:** 2022-11-10

**Authors:** Bart L. Clarke

**Affiliations:** 1 Mayo Clinic Division of Endocrinology, Diabetes, Metabolism, and Nutrition Rochester Minnesota USA Mayo Clinic Division of Endocrinology, Diabetes, Metabolism, and Nutrition, Rochester, Minnesota, USA

**Keywords:** Hypoparathyroidism, hypocalcemia, nephrolithiasis, nephrocalcinosis, increased bone density

## Abstract

The 2022 International Task Force guidelines for chronic hypoparathyroidism will be published within several months in the Journal of Bone and Mineral Research. These guidelines update the original guidelines published in 2016, and include new information from literature published since then. Chronic postsurgical hypoparathyroidism is now defined as lasting for at least 12 months after surgery, rather than 6 months. Chronic postsurgical hypoparathyroidism may be predicted by serum PTH <10 pg/mL in the first 12-24 hours after surgery. The most common symptoms and complications of chronic hypoparathyroidism based on the literature are summarized in detail. How to monitor and manage patients with hypoparathyroidism is described in detail where recommendations can be given. These guidelines are intended to frame the diagnosis and care of patients with chronic hypoparathyroidism for at least the next five years.

## INTRODUCTION

Care of patients with parathyroid disorders is often challenging due to complexities in the diagnosis, imaging, and surgical or medical management of primary hyperparathyroidism, and inadequacies with conventional management of hypoparathyroidism. This review summarizes recommendations from the 2^nd^ International Guidelines for Hypoparathyroidism (1) that will soon be published in the *Journal of Bone and Mineral Research*, along with the supporting systematic and narrative reviews (2–5). These guidelines summarize information published in the medical literature back as far as 1940, with particular focus on papers published between 1970 and 2020, and emphasizing new information published between 2015 and 2020. The previous 1^st^ International Guidelines on Hypoparathyroidism were published in 2016 (6).

## MATERIALS AND METHODS

The 2^nd^ International Guidelines on Hypoparathyroidism will be published in a separate upcoming issue from the 5^th^ International Guidelines on Primary Hyperparathyroidism in the *Journal of Bone and Mineral Research*. The 2^nd^ International Guidelines discuss the prevention, diagnosis, and management of hypoparathyroidism (HypoPT), and provide evidence-based recommendations for care of patients with this rare disorder. The four HypoPT Task Forces included a total of 50 international experts, including representatives of sponsoring international, national, and regional endocrine and medical societies. Dr. Guyatt and his team supported these taskforces and conducted the systematic reviews (7). A formal process following GRADE methodology and the systematic reviews provided the structure for 7 of the guideline recommendations. The Task Forces used a less structured approach based on narrative reviews with non-GRADEd recommendations for 20 of the recommendations.

Co-chairs of four Task Forces for HypoPT worked entirely virtually over an 18-month period due to the COVID-19 pandemic. Meetings were held regularly with specific tasks designated to individuals or subsets of Task Force members. A comprehensive review of the literature was undertaken by each of the Task Forces using the search engines described in the papers, including PubMed, Medline, Embase, and Cochrane.

Searches were conducted for systematic reviews, meta-analyses, and original publications. References to this field extended to 1940 for historical reference, but more recently between 1970 and 2020 for all other aspects of HypoPT, with particular emphasis on papers published between 2015-2020. For questions specifically related to medical management of HypoPT, GRADE methodology was employed (8). GRADEd recommendations followed a structured process that included framing questions in patient/intervention/comparator/outcome format; conduct of a systematic evidence search and associated summary; specification of values and preferences; and classifying and presenting recommendations as strong or weak with the corresponding quality of evidence. A strong recommendation was made when the desirable effects were much greater than undesirable effects or *vice versa*. The word “recommend” was applied to systematic reviews that reached this conclusion. A weak recommendation was made if there was low certainty of evidence or a close balance between desirable and undesirable effects. The word “suggest” was applied to systematic reviews that reached this conclusion.

Recognizing that this rigorous approach to evidence-based review may have necessarily omitted worthy observations due to the strict screening criteria for selection, the evidence from the GRADE methodology was amplified to incorporate other noteworthy observations. When recommendations based upon narrative reviews were made, the terms “the Panel recommends” or “the Panel proposes” or “Panel Recommendations (ungraded)” clearly distinguish ungraded recommendations from recommendations based upon the systematic reviews.

The Steering Committee organized the Task Forces with two co-chairs designated for each of the workshops. In collaboration with these Task Force co-chairs, 9-12 Task Force members were appointed to each Task Force. After drafts of each paper were prepared, a virtual meeting was held between the PHPT and HypoPT Task Force members. A second virtual meeting was then held, attended by representatives from all societies, organizations, and patient advocacy groups that expressed interest in this work with a view towards endorsing the guidelines. Recommendations from both meetings, each attended by about 100 individuals, were considered in revising and finalizing the papers that form the basis for the summary statements and their integration into this summary statement. The document was circulated to all organizations during a 6-8-week comment period. The organizations endorsing these guidelines will be listed at the end of the reference sections of the guidelines.

### Summary of recommendations for hypoparathyroidism

The recommendations summarized below are intended to guide practice and not intended to be used for the development of reimbursement policies for patients treated for hypoparathyroidism. Other than panel statements 3, 5, and 6 regarding surgical management of patients with primary hyperparathyroidism, which were based upon GRADE analysis, all the other panel statements either could not be analyzed by GRADE methodology (statements 1-2) or the available data were not of sufficiently high quality to permit GRADE analysis (statements 4, 7, 8) (7).

#### 1. How should chronic HypoPT be diagnosed?

Hypocalcemia is defined as low ionized serum calcium or total serum calcium adjusted for albumin in the presence of an undetectable or inappropriately low intact PTH measured with either a 2^nd^ or 3^rd^ generation assay on two occasions at least two weeks apart (6). This definition will eliminate misdiagnosis caused by a single measurement of low calcium due to other causes.

Additional abnormalities caused by low PTH supporting the diagnosis include increased serum phosphorus, decreased 1,25(OH)_2_D, and increased urinary fractional excretion of calcium (6). Based on discussions with surgeons on the task forces, postsurgical HypoPT is now considered permanent if the HypoPT persists > 12 months after surgery, rather than 6 months after surgery.

#### 2. How can the risks of chronic postsurgical HypoPT be minimized?

The infrequent occurrence of postsurgical HypoPT is a major source of morbidity to patients undergoing anterior neck surgery. Avoidance of parathyroid autotransplantation during anterior neck surgery may reduce the risk of chronic postsurgical hypoparathyroidism (9). Autotransplantation is recommended only in the setting of inadvertent parathyroidectomy (3).

#### 3. What is the value of determining serum calcium and PTH post-thyroidectomy to predict future permanent postsurgical HypoPT? (GRADEd Recommendation)

Serum PTH measurement after total thyroidectomy may be used to predict which patients will *not* develop permanent postsurgical HypoPT (strong recommendation, moderate quality evidence) (3). If PTH values are > 10 pg/mL (1.05 pmol/L) 12-24 hours after surgery, the development of permanent HypoPT is unlikely and, therefore, there is no need for long-term treatment with active vitamin D and calcium supplements above the recommended daily allowance. Many patients with PTH values < 10 pg/mL (1.05 pmol/L) 12-24 hours after surgery may recover from their temporary HypoPT.

#### 4. What is the role of genetic testing in the diagnosis and evaluation of chronic HypoPT?

In patients with nonsurgical HypoPT who have a positive family history of nonsurgical HypoPT or present with syndromic features, or are younger than 40 years, genetic testing is recommended (3,4,10). In patients with nonsurgical HypoPT who have other clinical features of autoimmune polyendocrinopathy-candidiasis-ectodermal dystrophy (APECED) syndrome, genetic testing for *AIRE* variants is recommended. The designation of “autoimmune HypoPT” for patients who do not have APECED syndrome should be avoided, as there are no definitive diagnostic tests for polygenic autoimmune HypPT (3).

#### 5. What are the most common symptoms and complications of chronic HypoPT reported in the literature? (GRADed recommendation)

Observational studies comparing patients with HypoPT to controls with normal parathyroid function have identified multiple complications associated with HypoPT (11–17). Percentages listed here represent the median among published studies. Cataracts are reported in 24% of patients, infections in 18%, nephrocalcinosis/nephrolithiasis in 15%, renal insufficiency in 13%, seizures in 12%, depression in 11%, ischemic heart disease in 9%, and arrhythmias in 7% (11). Other complications commonly associated with HypoPT, such as basal ganglia calcification, are not frequently reported in published case series, so are not included in the eight most common complications listed here.

#### 6. What is the optimal monitoring strategy for chronic HypoPT?

All panel members were surveyed regarding their current clinical practice, with 70% of respondents reported monitoring 70% of the time as described below (18). These recommendations are graded as low-quality since they are based on expert experience and opinion. The panel agreed that new patients should always be assessed for serum ionized or albumin-adjusted serum calcium, phosphorus, magnesium, creatinine, eGFR if creatinine clearance is not measured separately, 25OHD, and 24-hour urine calcium and creatinine. The panel also agreed that stable patients should be assessed for serum ionized or albumin-adjusted calcium, phosphorus, magnesium, creatinine, and eGFR every 3-12 months. Serum 25OHD should be remeasured every 6-12 months. 24-hour urine calcium and creatinine should be remeasured every 6-24 months. The consensus was that unstable patients should be assessed with frequent serum calcium and phosphorus measurements as clinically indicated.

The panel also proposed that patients should have baseline assessment for the presence of renal calcification or stones with renal imaging (11), and that serum ionized or albumin-adjusted calcium should be reassessed within several days of a significant change in medical treatment. These latter two recommendations were based on expert opinion and not survey results.

#### 7. How are patients with HypoPT managed? (GRADed recommendations)

In patients with chronic HypoPT, the panel suggests conventional therapy as first line therapy (weak recommendation, low quality evidence) (5,19,20). When conventional therapy is deemed unsatisfactory, the panel considers use of PTH (23). An insufficient number of placebo-controlled trials with PTH analogues has been published to date to justify a stronger recommendation.

#### 8. Recommendations for management:

In patients with chronic HypoPT, the panel proposed treatment with calcium and an active vitamin D analogue, with the goal of raising serum calcium into the target range, i.e., the lower half of the normal reference range or just below the normal reference range (5,19,20). At this time, it is not clear how to best balance the doses of calcium relative to those of the active vitamin D analogue. The goal should be to alleviate symptomatic hypocalcemia while avoiding hypercalciuria, and to avoid hypercalciuria when titrating calcium and active vitamin D analogue therapy, aiming for low-normal serum calcium levels.

The panel proposed achieving 24-hour urinary calcium of < 6.25 mmol or 250 mg for adult women and < 7.5 mmol or 300 mg for adult men, respectively. Data from the general population has shown a relationship between hypercalciuria and the development of renal stones, but such data does not exist yet in patients with HypoPT. However, panel members inferred that hypercalciuria may be associated with a higher risk of renal stones in patients with HypoPT as well, and thus proposed avoiding hypercalciuria. Hypercalciuria may be treated with thiazide diuretics in conjunction with a low sodium diet with careful monitoring of blood pressure, serum magnesium, potassium, and renal function (10).

Panel members proposed avoiding hyperphosphatemia. Panel members prescribe calcium supplements with meals to reduce phosphorus absorption after the meal, implement a low-phosphate diet in adults if needed, and judiciously use active vitamin D therapy that may increase phosphorus absorption. No data are available on the use of non-calcium phosphate binders, such as sevelamer, lanthanum, or others in HypoPT. Hyperphosphatemia may be associated with an increased incidence of ectopic calcification in other populations, but currently there is no evidence of this in HypoPT.

Panel members recommend treating to normalize serum magnesium levels. Magnesium supplements may be used as tolerated by the patient.

The serum 25OHD level should be kept in the normal reference range of the laboratory used (e.g., 20 to 50 ng/mL or 75-125 nmol/L).

PTH replacement therapy should be considered in patients who are not adequately controlled on conventional therapy. Inadequate control is considered to be defined when any one of the following are present despite maximal effort with conventional therapy: symptomatic hypocalcemia, hyperphosphatemia, renal insufficiency, hypercalciuria, or poor quality of life (21–31).

Individuals with poor compliance, malabsorption, or who are intolerant of large doses of calcium and active vitamin D may also benefit from PTH therapy. Individuals requiring high doses of conventional therapy, such as calcium supplement of > 2 mg/day, or active vitamin D > 2 mcg/day, may also benefit from PTH therapy (21).

#### 9. Ungraded Consensus Management Recommendations for Hypoparathyroidism During Pregnancy and Lactation

In pregnant women with HypoPT, the panel proposed that serum ionized or albumin-adjusted calcium should be maintained in the mid- to low-normal reference range throughout pregnancy (32–37). Serum phosphorus, magnesium, and 25OHD levels should be maintained in the normal reference range. Serum ionized or albumin-adjusted calcium should be monitored every 3-4 weeks during pregnancy and lactation, with increased frequency in the month preceding and following parturition, as well as in the presence of symptoms of hypercalcemia or hypocalcemia. Monitoring physicians should work closely with the obstetrician to optimize pregnancy outcomes, and coordinate with the neonatal pediatric team to ensure appropriate post-natal monitoring for transient hypo- or hypercalcemia in the infant. Thiazide diuretics, high-dose vitamin D2 or D3, and PTH or PTH analogues should be avoided during pregnancy.

## DISCUSSION

The 2^nd^ International Guidelines for Hypoparathyroidism represent the most recent evidence-based update to the previously published 1^st^ International Guidelines in 2016. These guidelines summarize the evidence published since 1940, but focus on more recent evidence published since 2000, and emphasize newly published research since 2016. The 90 or so international experts in parathyroid disorders who formed the four task forces addressing various aspects of hypoparathyroid disease were drawn from a wide pool of clinicians and scientists who have published in this area over the last 20 years.

For the first time, the recommendations generated in response to important questions felt to be relevant to current management of this disorder were evaluated using GRADE methodology where possible. Several systematic reviews were written describing the latest evidence in support of the new guidelines, and multiple narrative reviews written to evaluate recent evidence in topic areas without sufficient evidence to use GRADE methodology. These guidelines, summarizing multiple manuscripts written in support of the guidelines, are under review for eventual publication in the *Journal of Bone and Mineral Research* in the Fall of 2022, and have been presented at several national meetings in the U.S. and elsewhere.

New evidence has not changed the definition of HypoPT but has led to recommendation for biochemical assessment on at least two occasions using a 2^nd^ or 3^rd^ generation PTH assay. Most clinical hospital laboratories currently use a 2^nd^ generation assay. Patients with HypoPT are mostly moderately to severely symptomatic at presentation, but milder cases or those with longer-standing disease may be relatively asymptomatic. Those who are symptomatic commonly have had anterior neck surgery in the recent past, mostly for thyroid cancer, other head or neck cancer, or PHPT. Surveys have shown that about 75-80% of patients with chronic hypoparathyroidism have postsurgical hypoparathyroidism. Most of the postsurgical symptomatic patients will not have had end-organ involvement yet due to the short duration of their disease. Those with nonsurgical chronic hypoparathyroidism frequently have end-organ involvement due to the longer duration of their disease, sometimes for their entire lifetime if they have genetic disease. Patients with normocalcemic hypoparathyroidism have been described, but these clearly compose a small subset of the relatively asymptomatic group that may not have organ involvement and, by definition, have normal serum ionized and albumin-adjusted total calcium, but inappropriately low PTH levels. It is not yet clear that these patients benefit from specific treatment, and the guidelines do not make a recommendation for treatment of these mildly affected patients.

Recommendations for biochemical evaluation have not changed significantly. Measurement of serum calcium, phosphorous, creatinine, magnesium, intact PTH, and 25OHD, and 24-hour urine calcium and creatinine are advised. The 2^nd^ International Guidelines for Hypoparathyroidism give more precise recommendations for maintenance of 24-hour urine calcium in patients with this disorder at < 250 mg in women and < 300 mg in men. Earlier guidelines either did not specify a threshold for maintenance of 24-hour urine calcium or recommended keeping 24-hour urine calcium less than 400 mg to prevent kidney stones. The earlier threshold of 400 mg was recommended based on guidelines for prevention of kidney stones in the general population, and not specifically in patients with PHPT.

Skeletal assessment of patients with chronic HypoPT by bone density testing by DXA, vertebral fracture analysis, spine x-rays, or trabecular bone score, if available, are not routinely recommended because these patients usually have high bone mass due to absence or lack of circulating PTH. This leads to low bone turnover and relative preservation of bone mass. If DXA is to be used to assess bone density, it should be measured at the lumbar spine, total hip, and femur neck, and may be used at the 1/3 distal radius, to quantify the severity of bone loss. Patients with chronic HypoPT may have comorbidities that cause bone loss independent of their low serum PTH. Postmenopausal women may sometimes lose bone density despite being relatively protected against this.

Renal imaging by kidney x-ray, ultrasound, or CT are recommended to detect kidney stones or nephrocalcinosis, if present. Calcium-containing kidney stones may develop in patients with chronic HypoPT due to hypercalciuria that frequently develops due to high-dose calcium and/or active vitamin D supplements given over many years of treatment.

Medical management options are limited but may be useful in particular situations. Oral or intravenous bisphosphonates, denosumab, estrogen, and raloxifene should not be routinely used in patients with chronic HypoPT because these antiresorptive agents will cause the already-present low bone turnover to worsen. Cinacalcet should never be used as this prevents residual PTH secretion by the remaining parathyroid glands if present. Thiazide-type diuretics may be used to reduce urinary calcium excretion and prevent hypercalciuria and kidney stones.

These guidelines summarize current concepts regarding management of pregnancy and lactation in patients with HypoPT. Patients with HypoPT are at greater risk of adverse outcomes for themselves and their babies. Babies may develop postpartum hypercalcemia due to low maternal serum calcium if this is not adequately managed during pregnancy. Babies may also develop postpartum hypocalcemia if their mothers are over-treated with calcium and active vitamin D supplements during pregnancy.

New features of the 2^nd^ International Guidelines on Hypoparathyroidism include defining chronic postsurgical HypoPT as persisting for >12 months after anterior neck surgery. The previous guidelines (6) required that low serum calcium and parathyroid levels be present for at least 6 months to define chronic hypoparathyroidism. The duration of hypocalcemia and hypoparathyroidism was changed because occasional patients are able to recover from their postsurgical hypoparathyroidism for up to 12 months after surgery. Surgeons on the HypoPT task forces indicated that recovery from hypoparathyroidism may rarely occur later than 12 months after surgery.

To predict development of permanent postsurgical HypoPT, the guidelines recommend measuring serum PTH within 12-24 hours post-total thyroidectomy (strong recommendation, moderate quality evidence). PTH > 10 pg/mL (>1.05 pmol/L) is considered to virtually exclude long-term postsurgical HypoPT.

Patients with nonsurgical HypoPT may benefit from genetic testing in the presence of a positive family history of nonsurgical HypoPT, syndromic features, or in individuals younger than 40 years. The AIRE gene is frequently mutated in patients with nonsurgical chronic HypoPT. A variety of very rare mutations have been described that limit parathyroid function or prevent parathyroid gland development. Commercial laboratories offer gene panels in patients with idiopathic hypoparathyroidism.

HypoPT may be associated with multiple complications including nephrocalcinosis, nephrolithiasis, renal insufficiency, cataracts, seizures, cardiac arrhythmias, ischemic heart disease, depression. and increased risk of infection. Other complications are described in the literature, but some complications are less common or not routinely assessed for, and therefore not captured in the systematic review. Minimizing or preventing complications of HypoPT requires careful evaluation and routine monitoring of laboratory indices and imaging studies.

Patients with chronic HypoPT should be treated with conventional therapy with calcium and active vitamin D metabolites as first line therapy (weak recommendation, low quality evidence). Clinical experience has shown that many patients with chronic HypoPT are not adequately controlled with conventional therapy, despite the best efforts of knowledgeable and engaged clinicians. When conventional therapy is inadequate, use of recombinant human PTH is recommended. Multiple new agents are coming that will expand therapeutic options for treating HypoPT. These include TransCon PTH, other long-acting forms of PTH, oral teriparatide, PTH1 receptor agonists, and calcium-sensing receptor antagonists.

These guidelines also include ungraded consensus recommendations for management of pregnancy and lactation in patients with HypoPT. These are based on expert opinion due to lack of evidence beyond what has been published in small case series and case reports of women during pregnancy or lactation. No clinical trials have been conducted in women with HypoPT who are pregnant or lactating.

In conclusion: the 2^nd^ International Guidelines on Hypoparathyroidism provide updated evidence-based management recommendations for patients with chronic HypoPT, evaluated using GRADE methodology where possible. The 18-month effort behind these guidelines involved around 50 international experts in this disorder, as well as Dr. Gordon Guyatt and his methodology team from McMaster University in Canada. These guidelines will hopefully help advance the care of patients with HypoPT throughout the world
